# Zika Virus: the Latest Newcomer

**DOI:** 10.3389/fmicb.2016.00496

**Published:** 2016-04-19

**Authors:** Juan-Carlos Saiz, Ángela Vázquez-Calvo, Ana B. Blázquez, Teresa Merino-Ramos, Estela Escribano-Romero, Miguel A. Martín-Acebes

**Affiliations:** Department of Biotechnology, Instituto Nacional de Investigación y Tecnología Agraria y AlimentariaMadrid, Spain

**Keywords:** Zika, flavivirus, outbreak, microcephaly, zoonosis

## Abstract

Since the beginning of this century, humanity has been facing a new emerging, or re-emerging, virus threat almost every year: West Nile, Influenza A, avian flu, dengue, Chikungunya, SARS, MERS, Ebola, and now Zika, the latest newcomer. Zika virus (ZIKV), a flavivirus transmitted by *Aedes* mosquitoes, was identified in 1947 in a sentinel monkey in Uganda, and later on in humans in Nigeria. The virus was mainly confined to the African continent until it was detected in south-east Asia the 1980’s, then in the Micronesia in 2007 and, more recently in the Americas in 2014, where it has displayed an explosive spread, as advised by the World Health Organization, which resulted in the infection of hundreds of thousands of people. ZIKV infection was characterized by causing a mild disease presented with fever, headache, rash, arthralgia, and conjunctivitis, with exceptional reports of an association with Guillain–Barre syndrome (GBS) and microcephaly. However, since the end of 2015, an increase in the number of GBS associated cases and an astonishing number of microcephaly in fetus and new-borns in Brazil have been related to ZIKV infection, raising serious worldwide public health concerns. Clarifying such worrisome relationships is, thus, a current unavoidable goal. Here, we extensively review what is currently known about ZIKV, from molecular biology, transmission routes, ecology, and epidemiology, to clinical manifestations, pathogenesis, diagnosis, prophylaxis, and public health.

## The Virus

Zika virus (ZIKV) is an arbovirus (arthropod-borne virus) classified into the *Flavivirus* genus within the *Flaviviridae* family^1^. Flaviviruses are small enveloped single stranded positive RNA viruses that include important human and animal pathogens such as yellow fever virus (YFV), dengue virus (DENV), West Nile virus (WNV), St. Louis encephalitis virus (SLEV), Japanese encephalitis virus (JEV) or tick-borne encephalitis virus (TBEV) (Gould and Solomon, 2008). Historically, ZIKV was discovered in the course of investigations designed to study the vector responsible for the non-human cycle of yellow fever in Uganda almost 70 years ago. The first isolation was made in April 1947 from the serum of a febrile sentinel rhesus monkey (named Rhesus 766) that was caged in the canopy of Zika Forest, near Lake Victoria (Dick et al., 1952). The second isolation was made from *Aedes africanus* mosquitoes caught in the same forest in January 1948 (Dick et al., 1952). Thus, ZIKV received its name from the geographical area where the initial isolations were made. Both isolations were performed by intracerebral inoculation into *Swiss* albino mice of the samples containing the virus (serum from febrile monkey or mosquito homogenates) demonstrating that ZIKV was a filterable transmissible agent (Dick et al., 1952). These early filtration studies indicated that the size of ZIKV was in the range of about 30–45 nm in diameter (Dick, 1952). Further transmission electron microscopy analysis of ZIKV infected cells revealed that the virions were spherical particles with an overall diameter of 40–43 nm and a central electron dense core being 28–30 nm in diameter (Bell et al., 1971; Hamel et al., 2015). Although there are still no specific studies on the structure of ZIKV, it can be inferred from other flaviviruses (Mukhopadhyay et al., 2005) that the viral particles should be about 50 nm in diameter, which is compatible with the observations performed for ZIKV. Cryoelectron microscopy reconstructions of flavivirus particles have shown that virions are composed by a central core that contains the capsid or core (C) protein associated with the viral genomic RNA. This nucleocapsid is enclosed into a lipid bilayer derived from the host cell. The membrane (M) and envelope (E) proteins are anchored into the lipid envelope and conform the smooth outer shell of the virion, which is constituted by 180 copies of the M and E proteins arranged as 90 anti-parallel homodimers (Kuhn et al., 2002; Mukhopadhyay et al., 2003). Regarding the stability of the virion, it has been described that ZIKV suspensions were most stable at pH of 6.8–7.4 and particles were inactivated at pH of under 6.2 and over 7.8, by potassium permanganate, ether, and temperatures of 58 °C for 30 min, or 60°C for 15 min, but the infectivity was not effectively neutralized with 10% ethanol (Dick, 1952).

### Genome

**FIGURE 1:**
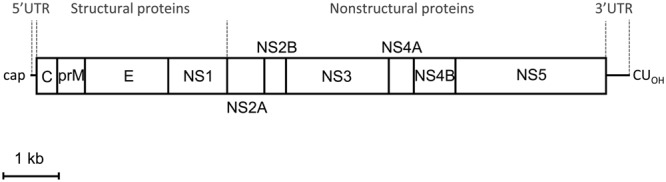
**Schematic view of Zika virus (ZIKV) genome organization.** The open reading frame (ORF) (boxes) that encodes structural and non-structural proteins is flanked by two untranslated regions (UTR). The proportion of each region was calculated from the ZIKV MR766 sequence available at GenBank (NC_012532.1). Scale bar: 1 kb.

The flavivirus genome is constituted by a single-stranded RNA molecule of positive polarity that, in a similar manner to cellular mRNAs, includes a cap structure at its 5′ end (Dong et al., 2014). Proper methylation of this structure is important not only for efficient translation of viral genome, but also for evasion of immune response (Daffis et al., 2010). The sequence of the prototype strain of ZIKV MR766, which corresponds to a passaged virus derived from the initial ZIKV isolated by intracerebral inoculation of the serum of the febrile monkey (Rhesus 766) into mice in 1947 (Dick, 1952; Dick et al., 1952), revealed that the ZIKV genome was 10794 nucleotides in length (Kuno and Chang, 2007). The genome contains a single open reading frame (ORF) that encodes a polyprotein of about 3400 amino acids (**Figure 1**) that is expected to be cleaved into the mature viral proteins (see next section for polyprotein processing). The single ORF is flanked by two untraslated regions (UTR) located at the 5′ and 3′ ends of the genome, which in the prototype ZIKV MR766 are of 106 and 428 nucleotides in length, respectively (Kuno and Chang, 2007). Remarkably, and in contrast to cellular mRNAs, ZIKV genome lacks a 3′ poly(A) tract and ends with CU_OH_ in a similar manner to the other flaviviruses. Subsequent studies have confirmed that this basic organization is shared among other isolates of ZIKV, although differences in length and nucleotide sequence have been documented among different isolates, even among ZIKV MR766 isolates with different passage history (Lanciotti et al., 2008; Haddow et al., 2012; Baronti et al., 2014; Berthet et al., 2014). The cyclization of flavivirus genome between 5′ and 3′ terminal regions, which is important for the functionality of the genome, is mediated by the interaction of complementary sequences located with genome regions termed conserved sequences (CSs). These CS (CS1 to CS3) are also present in the ZIKV genome, suggesting that has the potential for cyclization. Nevertheless, it has to be remarked that the organization of the CS in the 3′end of ZIKV is different from that of other mosquito-borne flaviviruses (Kuno and Chang, 2007).

### Viral Proteins

The viral polyprotein encoded by the single ORF in ZIKV (**Figure 1**), as in other related flaviviruses, is supposed to be cleaved by cellular and viral proteases into three structural proteins: the capsid (C), premembrane/membrane (prM/M), and envelope (E), and seven non-structural proteins (NS1, NS2A, NS2B, NS3, NS4A, NS4B, and NS5). The predicted cleavage sites of ZIKV basically follow the patterns established for other mosquito-borne viruses, and, likewise, cysteine residues within the polyprotein are well conserved relative to other mosquito-borne flavivirus (Kuno and Chang, 2007). Different proteases participate in the processing of the viral polyprotein: the host cellular signalase which cleaves M/E, E/NS1, and the C-terminal hydrophobic region of NS4A (termed 2K peptide)/NS4B. The viral serine protease (NS3) is expected to cleave the junctions between the virion capsid protein (C_v_) and the C-terminal hydrophobic domain of capsid protein (C_i_) [C_v_/C_i_], NS2A/NS2B, NS2B/NS3, NS3/NS4A, NS4A/2K peptide, and NS4B/NS5. The NS1/NS2A is believed to be cleaved by an unknown cellular signalase (Kuno and Chang, 2007). Of key importance is the proteolytic cleavage of prM to give the pr peptide and M protein, which is produced by furin-like protease located in the *trans*-Golgi network during the egress of the particles and promote the maturation of the virions (Mukhopadhyay et al., 2005).

The analysis of the polyprotein sequence predicts the presence of potential N-glycosylation sites in the ZIKV proteins prM, E, and NS1 (Kuno and Chang, 2007; Baronti et al., 2014; Berthet et al., 2014). However, the functional significance of the *N*-glycosylations is not clear in related flaviviruses, since deglycosylated flaviviruses can maintain the same antigenicity, suggesting that carbohydrate does not play a major role in the antigenic properties of the virus (Winkler et al., 1987), and that glycosylation does not alter epitope recognition (Vorndam et al., 1993). On the other hand, glycosylation could be important for replication and maturation (Li et al., 2006). In the case of ZIKV, there are differences among strains due to a 12 nucleotides deletion on the glycosylation motif located at position 154 in the E protein (E-154), which is present in many flaviviruses (Lanciotti et al., 2008; Haddow et al., 2012; Baronti et al., 2014). Remarkably, there are differences on this site even between ZIKV isolates with different passage history, such as those of the prototypic strain ZIKV MR766, indicating that passage history influences glycosylation sites (Haddow et al., 2012). However, the loss of glycosylation on the E-154 residue is not unique to ZIKV and has also been observed in other flaviviruses (Adams et al., 1995; Berthet et al., 1997). Although the functional role of glycosylation in the E protein is not clear, the presence of this glycosylation in other flaviviruses has been associated with the ability to cause significant human outbreaks (Shirato et al., 2004). Moreover, it has been suggested that the fact that ZIKV strains isolated during the recent human outbreak in Oceania contain this N-linked glycosylation signal, whereas the majority of other strains does not, could indicate that the N-linked glycosylation of the E protein plays a role on the pathogenicity of ZIKV (Baronti et al., 2014; Berthet et al., 2014). Nevertheless, functional studies are required to provide an experimental confirmation of this hypothesis.

Regarding the functions of the flaviviral proteins, the three structural proteins participate in the assembly of the virions. As commented above, the C protein associates with the genomic RNA to conform the core of the virions, and the E protein should mediate the binding to the cellular receptor of the virus and promotes the fusion of the virions with the endosomal membranes of the target cell during viral entry (Mukhopadhyay et al., 2005; Roby et al., 2015). Relative to prM function, this protein assists the folding of E protein as a sort of chaperone and prevents premature fusion of the particles prior to be released from the infected cell, and the cleavage of prM into M protein also promotes the maturation of the viral particles (Mukhopadhyay et al., 2005; Roby et al., 2015).

To our knowledge there are currently no specific studies addressing the function of non-structural proteins of ZIKV, but it is expected that some functions could be inferred from related flaviviruses (Martin-Acebes and Saiz, 2012; Acosta et al., 2014), such as the induction of membrane rearrangements associated with flavivirus replication (NS4A), and the immunomodulation (NS1, NS2A) or regulation of RNA replication and viral assembly (NS2A). Furthermore, NS2B acts as a cofactor for the viral trypsin-like serine protease NS3, which can also act as a helicase. NS5 is the viral RNA dependent RNA polymerase that is in charge of genome replication, and that also displays a methyltransferase domain necessary for capping the 5′ end of the viral genomic RNA. Since flavivirus non-structural proteins constitute major targets for antiviral research (Noble and Shi, 2012; Luo et al., 2015), deciphering the specific role of the non-structural proteins in ZIKV infection could greatly contribute to the development of antiviral strategies against this pathogen.

### Host Cell-Virus Interactions

Zika virus can infect a broad range of cells from different tissues and species. For instance, experimental infection by blood meal has revealed that ZIKV replicates in the midgut and salivary glands of diverse *Aedes* mosquitoes (Li et al., 2012; Wong et al., 2013), and also *in vitro* in cultured mosquito cells C6/36 (Hamel et al., 2015). ZIKV also replicates in a wide variety of mammalian cell types. Experimental infection in mice has revealed that the virus replicates mainly in brain cells, including neurons, and astroglial cells (Weinbren and Williams, 1958; Bell et al., 1971), and, *in vitro*, it can replicate in cultured monkey cell lines such as LLC- MK2, or Vero, inducing cytopathic effect (Way et al., 1976). Furthermore, the titer of ZIKV in cultured cells seems to well correlate with the infectivity of the virus *in vivo* (Way et al., 1976). In addition, it has also been recently reported that ZIKV can replicate in human skin cells and also in immature dendritic cells (Hamel et al., 2015). This ability of the virus to replicate in cells from different sources could be related to its transmission cycle, which includes replication in mosquito (vector) and mammalian cells (host).

**FIGURE 2:**
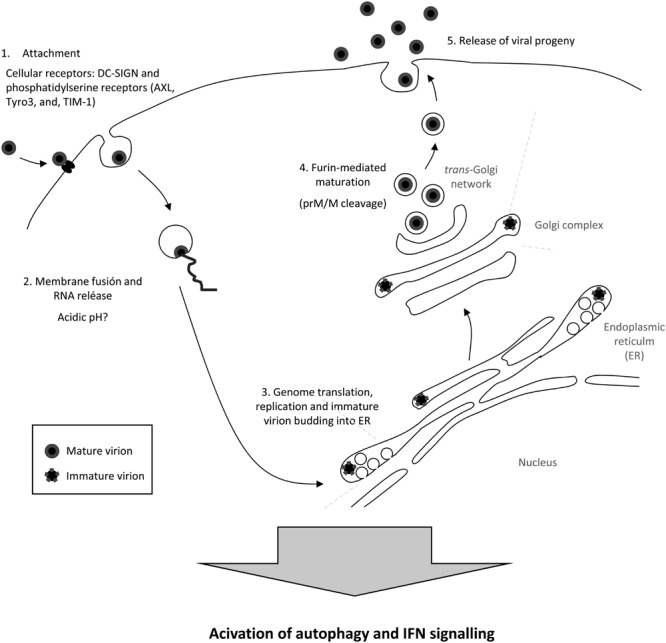
**Schematic view of the ZIKV cellular lifecycle.** See text for details.

It has been described that ZIKV enters the cell using adhesion factors such as DC-SIGN (Dendritic Cell-Specific Intercellular adhesion molecule-3-Grabbing Non-integrin) and diverse members of the phosphatidylserine receptor family (Hamel et al., 2015). Once the attached viral particles are internalized into the cell (**Figure 2**), the viral genome should be released inside the cytoplasm to start translation and replication. The mechanism of penetration of the flavivirus genome into the cytoplasm is initiated by the fusion of the viral envelope with the membranes of the cellular endosomes from the host cell, a process triggered by acidic pH inside cellular endosomes (Stiasny et al., 2011; Vazquez-Calvo et al., 2012). This mechanism of penetration is consistent with, as mentioned before, an early observation showing that ZIKV particles were sensible to acidic pH, and were inactivated by treatment with acidic pH lower than 6.2 (Dick, 1952). Along this line, the sensitivity of ZIKV particles to acidic pH is consistent with the observation performed with other flaviviruses indicating that, in the absence of target membranes, the exposure of flavivirus virions to acidic pH induces rearrangements of the E glycoprotein, which result in a loss of infectivity (Gollins and Porterfield, 1986). The viral RNA acts as mRNA inside the cytoplasm of the infected cell, and negative-strand viral RNA is synthesized and directs positive-strand RNA synthesis in association with a virus-induced network of membranes derived from the endoplasmic reticulum, ER (**Figure 2**). Electron microscopy studies of virus infected cells showed that ZIKV virions are found in short chains within tubular elements of the ER, which appeared to be in continuity with distended cisternae (Bell et al., 1971). These images were similar to membrane rearrangements observed in other flavivirus infected cells (Welsch et al., 2009; Martin-Acebes et al., 2011; Miorin et al., 2013). Although flavivirus replication is thought to occur in the cellular cytoplasm, it should be noted that one study reported that ZIKV antigens could be found in infected cell nuclei (Buckley and Gould, 1988).

*De novo* synthesized positive strand-RNA has to be packaged in progeny virions that bud into the ER to form enveloped immature virions (**Figure 2**). These virions traffic through the Golgi complex and, then, the prM is cleaved in the *trans*-Golgi network for particle maturation prior to release from the infected cell (Mukhopadhyay et al., 2005; Roby et al., 2015). Remarkably, the observations made for other flaviviruses have revealed that not all the copies of the prM protein in the secreted virions are cleaved, and, thus, that a proportion of them remains unprocessed in the secreted virions (Plevka et al., 2011). Even more, the amount of prM within the flavivirus virions varies with the cell line used for the production of the virus and the infecting flavivirus, and could be of key importance for the antigenicity of the particles (Pierson and Diamond, 2012; Lok, 2016).

Knowledge regarding the cellular response to ZIKV infection is still scarce. However, it has been experimentally probed that replication of ZIKV provokes an innate antiviral response, inducing the transcription of TLR3, RIG-I, and MDA5, as well as that of several interferon stimulated genes, such as OAS2, ISG15, and MX1, characterized by a strongly enhanced beta interferon gene expression (Hamel et al., 2015). In addition, ZIKV infection is sensitive to interferon (IFN) signaling, as pretreatment of primary skin fibroblasts with IFN-alpha, beta, and gamma reduces ZIKV infection (Hamel et al., 2015). ZIKV infection also upregulates the autophagic pathway in infected skin fibroblasts (Hamel et al., 2015), which is consistent with the observations for other related flaviviruses (Blazquez et al., 2014). Moreover, the autophagic marker LC3 colocalizes with viral proteins within ZIKV-infected cells, and the infection can be reduced by treatment with the autophagy inhibitor 3-methyladenine, whereas upregulation of autophagy using Torin 1 increases ZIKV replication (Hamel et al., 2015).

## Molecular Classification

**FIGURE 3:**
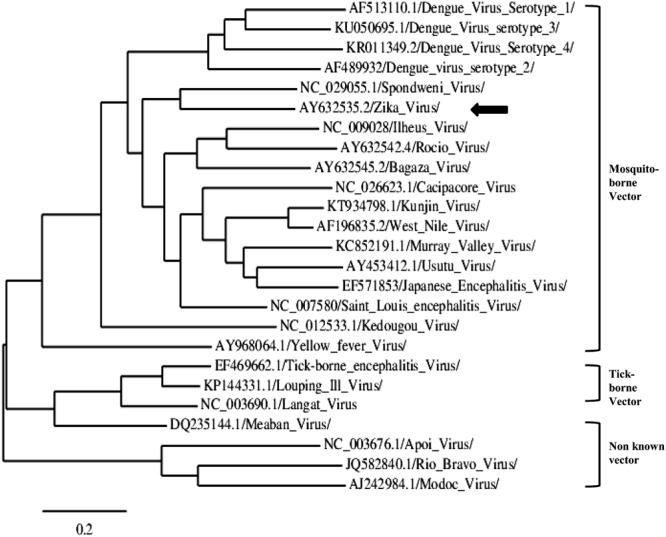
**Representative phylogram showing the relationships between strains of the genus Flavivirus.** Accession numbers are displayed in the tree. The arrow indicates ZIKV. The scale indicates 0.2 substitutions/site. The tree was based on complete NS5 nucleotide sequence, built from a multiple alignment using Clustal omega and Phylogeny.fr (Dereeper et al., 2008).

**FIGURE 4:**
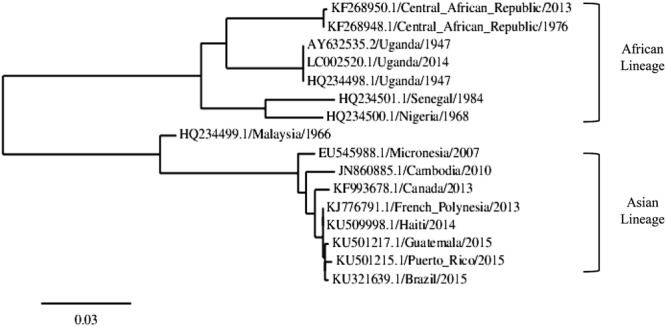
**Representative phylogram showing the relationships between ZIKV strains.** The accession number, country of isolation and year is displayed in the tree. The scale indicates 0.03 substitutions/site. The tree was based on complete NS5 nucleotide sequence, built from a multiple alignment using Clustal omega and Phylogeny.fr.

Zika virus is genetically and antigenically related to Spondweni virus. Both viruses form a unique clade (clade X) within the mosquito-borne flavivirus cluster (Kuno et al., 1998) (**Figure 3**). Phylogenetic analyses reveal the existence of two major lineages: one includes the African strains, and the other the Asian and American strains (Haddow et al., 2012; Alera et al., 2015) (**Figure 4**). The African lineage is further divided into two groups, the East African cluster, containing the genetic variants of the prototypic MR766 strain isolated in Uganda in 1947, and a second group including West African strains (Olson et al., 1981).

Phylogenetic studies (Faye et al., 2014) have established the date of the emergence of ZIKV in east Africa around 1920 (confidence range of 1892–1947). The same study dated the transmission of eastern African ZIKV to Asia around 1945 (confidence range 1920–1960), where the virus was first detected in the late 1960s in Malaysia (Marchette et al., 1969), and subsequently across south-east Asia. These data indicated a widespread occurrence of ZIKV from Africa to Southeast Asia, west and north of the Wallace line (Lanciotti et al., 2008).

Phylogenetic studies have confirmed that Pacific Island ZIKV strains are related to the Asian lineages (Gatherer and Kohl, 2016). Due to the great geographical distances involved, it seems likely that the virus was introduced to the island either by a viremic person, an enzootic host species, or an infected mosquito transported to the island (Haddow et al., 2012). On the other hand, recent transmission to the Americas appears to have originated in the Pacific Islands (Campos et al., 2015; Zanluca et al., 2015). Phylogenetic analysis of the sequences placed the Brazilian and other American strains in a clade with sequences from the Asian lineage, showing a 99% identity with a sequence from a ZIKV isolate from French Polynesia (Baronti et al., 2014; Campos et al., 2015) (**Figure 4**). It has been postulated that two events may have led to the introduction of ZIKV in Brazil, the 2014 FIFA World Cup tournament and an international canoe racing event (Musso, 2015), but since Pacific nations were only represented among the canoe racers, the latter seems to be a likeliest introduction route.

As exemplified in **Figure 4**, ZIKV strains collected in the same geographical region during several years show minimal changes on their sequences, as is the case of strains collected from mosquitoes in a 3 years interval in Central African Republic (Berthet et al., 2014). In this regard, it has been described that infection and transmission modes of ZIKV allow the accumulation of synonymous mutations and negatively selected certain sites (Faye et al., 2014). The arbovirus life cycle imposes several barriers to non-synonymous mutations in some important genes as a consequence of the intrinsic constraints associated with dual replication in mammalian and invertebrate hosts, thus driving to a more slowly fixation of mutations of these viruses when compared with RNA viruses transmitted by other routes. In fact, arboviruses, which are able to successfully adapt to diverse cell types, are characterized by a high rate of deleterious mutations (Holmes, 2003).

Human-to-human transmission of the Asian ZIKV strains along the Pacific Islands and South America has been associated with significant NS1 codon usage adaptation to human housekeeping genes, which could facilitate viral replication and increase viral titers (Freire et al., 2015). Furthermore, this report predicted the presence of several epitopes in the NS1 protein that are shared between ZIKV and DENV, pointing to a significant dependence of the recent human ZIKV spread on NS1 translational selection.

As mentioned above, it is noteworthy to note that several of the ZIKV strains exhibited a 4 amino acid deletion corresponding to the envelope protein 154 glycosylation motif found in many flaviviruses (Berthet et al., 2014).

## Transmission Cycle

### Mosquitoes

The arthropod vectors of the ZIKV natural transmission cycle are mosquitoes of the genus *Aedes* (Diagne et al., 2015). As mentioned early, the virus was first isolated from *A. africanus* (Dick et al., 1952) and, since then, ZIKV has been isolated from *A. aegypti* (Marchette et al., 1969), the main vector of the virus, and also from *A. albopictus* (Grard et al., 2014), confirming that both species are competent vectors.

*Aedes aegypti* is currently distributed in Asia and Oceania, the Americas, and in a few regions of Africa and Europe (Madeira and the north-eastern Black Sea coast)^2^; however, it has been recently predicted that this species might soon colonize some southern European regions, as well as temperate North America and Australia (Kraemer et al., 2015). The first evidence of the role of *A. aegypti* in the urban transmission cycle of ZIKV was suggested after its isolation from a pool of mosquitoes collected in 1966 in Malaysia, in what it was the first isolation from a mosquito other than *A. africanus* (Marchette et al., 1969).

*Aedes albopictus*, the so-called Asian tiger mosquito, is also widely distributed. This species is currently circulating in Asia, North, Central and South America (Kraemer et al., 2015), northern Australia, and in some areas of Africa and southern Europe, where it has spread in the past two decades to France, Germany, Italy, and Spain (Paupy et al., 2009; Dyer, 2016). Contrary to *A. aegypti, A. albopictus* can hibernate and survive in cool temperature regions (Thomas et al., 2012). This species can also efficiently transmit the virus, as demonstrated during the outbreak that took place in Gabon in 2007, where, among all species tested (including *A. aegypti*), it was the only one in which the virus was detected, thus confirming that *A. albopictus* may also play an important role in ZIKV transmission (Grard et al., 2014).

The ability of ZIKV to be efficiently transmitted by both mosquito species (*A. aegypti* and *A. albopictus*) that feed on humans further complicates their control and, thus, that of ZIKV. Both species grow very close to human populations, but while *A. aegypti* feed almost exclusively on humans in daylight hours and typically rest indoors (Scott and Takken, 2012), *A. albopictus* is usually exophagic and bites humans and also domestic and livestock animals (Paupy et al., 2009), although under some circumstances it preferentially feed on humans, hence, confirming that it can also have an anthropophilic behavior similar to *A. aegypti* (Ponlawat and Harrington, 2005; Delatte et al., 2010). Therefore, methods of control for a species may not be accurate to control the other one. Furthermore, when the populations of *A. aegypti* is reduced, the opportunistic invasive *A. albopictus* may rapidly move into the area (Higgs, 2016).

*Aedes aegypti* and, to a lesser extent, *A. albopticus* are clearly involved in ZIKV transmission and spread, but other *Aedes* spp., such as *A. polynesienis*, are suspected to have also contributed to it, as it was the case during the 2013–2014 outbreak in the French Polynesia (Cao-Lormeau et al., 2014). In fact, ZIKV has been also isolated from, at least, other 15 *Aedes* species in different regions of the world (**Table 1**). Even more, in the Kédougou region of Senegal, ZIKV was also amplified from pools of three mosquito species other than *Aedes: Anopheles coustani, Culex perfuscus*, and *Mansonia uniformis* (Diallo et al., 2014).

**Table 1:** Zika vector mosquito species.

Species	Arguments in favor of vector status	Place and year of first report (Reference)
*Aedes africanus*	Isolation	Zika Forest (Uganda), 1948 (Dick et al., 1952)
*Aedes aegypti*	Isolation	Malaysia, 1966 (Marchette et al., 1969)
*Aedes luteocephalus*	Isolation	Saboya Forest (Senegal), 1968 (Haddow et al., 2012; Diallo et al., 2014)
*Aedes vittatus*	Isolation	Senegal 1968 (Haddow et al., 2012)
*Aedes dalzieli*	Isolation	Senegal 1968 (Haddow et al., 2012)
*Aedes metallicus*	Isolation	Senegal 1968 (Haddow et al., 2012)
*Mansonia uniformis*	Isolation	Senegal 1968 (Haddow et al., 2012)
*Aedes fowleri*	Isolation	Senegal 1968 (Haddow et al., 2012)
*Aedes minutus*	Isolation	Senegal 1968 (Haddow et al., 2012)
*Aedes neoafricanus*	Isolation	Senegal 1968 (Haddow et al., 2012)
*Aedes tarsalis*	Isolation	Senegal 1968 (Haddow et al., 2012)
*Aedes apicoargenteus*	Isolation	Central African Republic, 1969 (Diagne et al., 2015)
*Aedes furcifer-taylori*	Isolation	Bandia (Senegal), 1969 (Diallo et al., 2014)
*Annopheles gambiae s.l.*	Isolation	Bandia (Senegal), 1969 (Diallo et al., 2014)
*Aedes opok*	Isolation	Central African Republic, 1976–1980 (Berthet et al., 2014)
*Aedes taylori*	Isolation	Kédougou region (Senegal), 1988–1991 (Monlun et al., 1993)
*Aedes albopictus*	Epidemiology	Gabon, 2007 (Grard et al., 2014)
*Aedes hirsutus*	Epidemiology	Kédougou region (Senegal), 2011, (Diallo et al., 2014)
*Aedes unilinaetus*	Epidemiology	Kédougou region (Senegal), 2011, (Diallo et al., 2014)
*Culex perfuscus*	Epidemiology	Kédougou region (Senegal), 2011, (Diallo et al., 2014)
*Anopheles coustani*	Epidemiology	Kédougou region (Senegal), 2011, (Diallo et al., 2014)

As in other arboviral infections, local overwintering could be an important aspect for maintenance and spread of ZIKV. Detection of the virus in a pool of *A. furcifer* males in 2011 in Senegal, even though no infected *A. furcifer* females were collected, strongly suggested that ZIKV is vertically transmitted, at least in this species, and that this transmission route may be an important mechanism of local maintenance (Diallo et al., 2014).

Finally, although most of these data were obtained by analyses of ZIKV naturally infected mosquitoes, their current vector competence still has to be clearly established. In this sense, different *Aedes* species (*A. aegypti, A. unilineatus, A. vittatus*, and *A. luteocephalus*) were recently tested in their susceptibility to ZIKV oral infection, and, although all of them were susceptible, viral genome could be amplified from saliva only in the case of *A. vittatus* and *A. luteocephalus* mosquitoes (Diagne et al., 2015).

### Humans and Non-human Primates

During outbreaks, humans are the primary host for ZIKV (Staples et al., 2016), and both urban (Grard et al., 2014) and sylvatic (Berthet et al., 2014) viral transmission have been demonstrated. First ZIKV isolation from humans was reported in 1954 in Nigeria (Macnamara, 1954), although antibodies to the virus had been previously found during early surveys on human sera in different regions of Africa (Dick, 1952; Smithburn, 1952). Recently, and albeit an estimated 80% of ZIKV infected people are asymptomatic^3^, during the ongoing outbreak in Brazil, ZIKV RNA has been identified in brain, placenta, and amniotic fluid specimens, and its presence has been associated to microcephaly in infants and miscarries during pregnancy (Martines et al., 2016; Mlakar et al., 2016).

One important aspect that still has to be clarified is whether ZIKV infection in humans drives to viral titers enough to initiate a new cycle when an infected person is bitten by a naïve mosquito. Early studies attempting to infect *A. aegypti* from a ZIKV-infected human volunteer to further transmit the agent to newborns mice were unsuccessful (Bearcroft, 1956), probably because viremia was too low, even though the volunteer was bitten during the acute phase of the disease (4–6 days after infection). Later on, a recent attempt to isolate circulating virus from infected patients of the 2007 Gabonese outbreak on mammalian (Vero) and insect (C6/36) cell lines was again unsuccessful, presumably because of low viral titers (despite two patients presenting only 1 and 4 days after symptom onset), although the inappropriate initial storage conditions could have also contributed to it (Grard et al., 2014). In this line, the estimated number of genome copies circulating in ZIKV-infected patients during the 2007 outbreak on the Pacific Island of Yap was reported to be 0.9 × 10^3^–7.2 × 10^5^ cDNA copies/ml (Lanciotti et al., 2008). These relatively low levels of viremia among ZIKV-infected patients are very far from those reported for other arboviruses, as Chikungunya (CHIK) and DENV-2, where an estimated 10^7^ and 10^8^ cDNA copies/ml were reported, respectively (Caron et al., 2012), but they are in the range of other dead-end flavivirus infection in humans, such as that of WNV, where viral loads from 50 to 6.9 × 10^5^ copies/ml are observed (Pupella et al., 2013). Therefore, further experiments are needed to clarify this issue.

In the case of non-human primates, it is known that epizootics occur in them (McCrae and Kirya, 1982), but it is unclear whether they are an obligatory reservoir in the transmission to humans. In Africa, ZIKV natural transmission cycle involves primarily *Cercopithecus aethiops* and *Erythrocebus patas* monkeys (Faye et al., 2013). In Asia (Borneo), antibodies against ZIKV have been detected among semi-captive and wild orangutans (Wolfe et al., 2001). However, this study reported a higher prevalence of anti-ZIKV antibodies in humans than in orangutans, suggesting a possible incidental infection of these animals through contact with mosquitoes infected by viremic people, or from recently established sylvatic cycles. Nonetheless, it is also possible that sylvatic ZIKV transmitting mosquitoes in Borneo have a more narrow distribution, or an ecology that does not lead to frequent exposure by orangutans.

An acute symptomatic ZIKV infection case after a monkey bite has been recently described (Leung et al., 2015). Even though transmission throughout mosquito bite could not be completely discharged, the presence of ZIKV in the human pharynx and the previous identification of ZIKV RNA in saliva from asymptomatic infected patients (Besnard et al., 2014) could be consistent with a potential transmission from primates’ bite (Leung et al., 2015).

### Other Vertebrates

Information regarding the possible susceptibility of animals other than human and non-human primates is limited. Antibodies directed against ZIKV have been found in several vertebrates species, such rodents, birds, reptiles, goats, sheep, and cattle in Kenya (Johnson et al., 1977), and in Pakistan, where some species of rodents were suggested as possible reservoirs of ZIKV (Darwish et al., 1983). In addition, the rapid periodicity of amplification observed in Senegal along the 2011 outbreak could support that, besides primates, other vertebrates may also play a role in ZIKV circulation (Diallo et al., 2014).

### Non-vector

Sporadic reports of direct human-to-human transmission have been reported to occur perinatally, sexually, and through breastfeeding and blood transfusion. Likewise, occupational transmission in the laboratory setting has also been described (Filipe et al., 1973).

Perinatal transmission from two mothers to their new-borns during the French Polynesia outbreak has been documented, although contamination during delivery was not completely discarded (Besnard et al., 2014). Sera from the mothers were positive for ZIKV by reverse transcriptase polymerase chain reaction (RT-PCR) within 2 days post-delivery and those of their newborns within 4 days after birth. In addition, high ZIKV RNA load was detected in breast milk samples from both mothers, albeit the virus could not be multiplied in susceptible cell cultures. Therefore, ZIKV transmission by breastfeeding must be considered and further clarify. Similarly, a first case of perinatal transmission of ZIKV was suspected to have occurred during the same outbreak from a mother that present a ZIKV infection-like syndrome 2 weeks before delivery, with the newborn showing a maculopapular rash at birth; however, virological investigations were not performed in this case (Besnard et al., 2014).

Besides these sporadic cases of non-vector transmission, the most surprising phenomenon on ZIKV infection is, without any doubt, the unexpected number of infants supposedly born with microcephaly (see clinical manifestations and pathogenesis section below) in Brazil during the ongoing viral outbreak, apparently as a result of their mothers being infected during pregnancy, since, in some cases, ZIKV-RNA has been detected in the amniotic fluid of the mothers^4^. Around 4000 cases of Zika-related microcephaly have been recorded in that country until now since October 2015, accounting for over 40 infants deaths (Higgs, 2016). Furthermore, ZIKV has been recently found in the fetal brain tissue of a baby with microcephaly after termination of the pregnancy requested by the infected woman (Mlakar et al., 2016).

At present (February 2016), three reported cases indicate that ZIKV could be sexually transmitted. In August 2008, a scientist was bitten many times while studying mosquitoes in south-eastern Senegal. Six days after returning to his home in Colorado (U.S.), he felt ill with symptoms of Zika fever and hematospermia. By then, he had had unprotected intercourse with his wife, who had not been outside the U.S. during the previous year, which subsequently developed symptoms of Zika fever. Virus infection was confirmed by serologic testing in both, but the presence of ZIKV in the semen of the patient was not investigated (Foy et al., 2011). A second report described the presence of replicative ZIKV and a high ZIKV RNA load in semen (1,1–2,9 × 10^7^ copies/ml) and urine (3,8 × 10^3^ copies/ml) samples of a patient during the 2013 outbreak in Tahiti, but no RT-PCR amplification was obtained from sera collected at the same time (Musso et al., 2015b). In early February 2016, the Dallas County Health and Human Services Department (U.S.) reported a third case, still under investigation, of a person that apparently contracted Zika fever after sexual contact with an ill person who had recently returned from a ZIKV high risk country^5^.

In addition to perinatal, breastfeeding, and sexual sporadic transmission of ZIKV, the potential for viral transmission through blood transfusion was demonstrated during the French Polynesian outbreak. Almost 3% (42/1505) of blood donors, who were asymptomatic at the time of donation, were found positive for acute ZIKV infection by specific RT-PCR (Musso et al., 2014a). Further studies are needed to assess the actual risk of ZIKV transmission through blood products and the risk to generate a disease in the recipient, but these data point to the necessity for quickly adapting blood donation safety procedures to the local epidemiological context. In fact, the Pan American Health Organization, PAHO^6^, and the European Centre for Disease Prevention and Controls, ECDC^7^, have recently issued a bulletins to alert their national health and blood safety authorities on this still poorly recognized viral infection.

Finally, ZIKV has been also detected in saliva samples (Besnard et al., 2014) with even higher frequency than in blood samples (Musso et al., 2015a) and, thus, saliva is another transmission source that have to be considered.

In resume, although ZIKV transmission by routes other than mosquito bites has been so far sporadic, further studies are mandatory to clearly establish their possible role on ZIKV epidemics.

## Ecology

Most arboviruses are perpetuated in transmission cycles independent of human hosts, but those with sylvatic cycles often infect people who accidentally intrude on their natural habitats. Nonetheless, in many instances, humans are dead-end hosts in complex transmission cycles that involve different wild and domestic vertebrate hosts, as in the case of JEV (Wolfe et al., 2001) or WNV (Martin-Acebes and Saiz, 2012). Less frequently, arboviruses may jump from this sylvatic transmission cycle to a mainly human-mosquito transmission cycle, as it likely was the case of CHIKV.

In the case of ZIKV, early studies indicated that non-human primates were the primary vertebrate hosts, with occasional participation of humans in the transmission cycle, even in highly enzootic areas. This theory was based on evidences indicating that *A. africanus*, a species with a greater preference for them than for humans (Haddow and Dick, 1948; Haddow et al., 1964), was the principal (if not the only) ZIKV vector (Haddow et al., 1964). However, by now, it seems clear that, as commented before, *A. aegypti* and, to a lesser extent, *A. albopticus* are the main vectors and, thus, that humans probably serve as primary amplification hosts when their viremia is sufficient in duration and magnitude (Haddow et al., 2012).

Although it is still unknown if ZIKV overwinters in geographical areas without reports of cases, it appears that, as in other flaviviral infections, such as that caused by WNV (Martin-Acebes and Saiz, 2012), epidemics are more related to the specific mosquito species, its population density, competence, and behavior in a given area, as this can shape virus dynamics.

*Aedes aegypti*, which, as mentioned before, is found throughout Asia, Oceania, the Americas and in some regions of Africa and Europe^2^ (Kraemer et al., 2015), does not overwinter, but can be sheltered in domestic settings, which provides protection against environmental conditions and numerous aquatic habitats suitable for oviposition. Beside Georgia, this species does not currently circulate in Europe, but there are no climatic reasons to believe that, if re-introduced, it cannot become widely established in southern Europe as before (Reiter, 2010).

Meanwhile, *A. albopictus*, the most invasive mosquito species in the world (Medlock et al., 2012), have spread during the last 30–40 years to North, Central and South America, parts of Africa, southeastern Asia, China, Japan, northern Australia, and southern Europe (Paupy et al., 2009). This successfully colonization of new regions is due to its ability to adapt to different climates through the production of cold-resistant eggs, with temperate strains surviving cold winters in northern latitudes. On top of that, its preference for container habitats (e.g., tires and vases) in domestic settings has resulted in increased potential for contact with humans (Medlock et al., 2012).

ZIKV pandemic is currently in progress, with many important questions still unanswered. However, as seen during recent years with other viral agents, urban overcrowding, constant international travel, disruption of the ecologic balance, and climate changes can favored the unexpected emergence of asleep, or yet unknown, infectious agents (Fauci and Morens, 2016). Therefore, comprehensive and integrated investigations have to be conducted to better understand the complex ecosystems in which agents of current and future pandemics could be aggressively evolving.

## Epidemiology

### Africa

**FIGURE 5:**
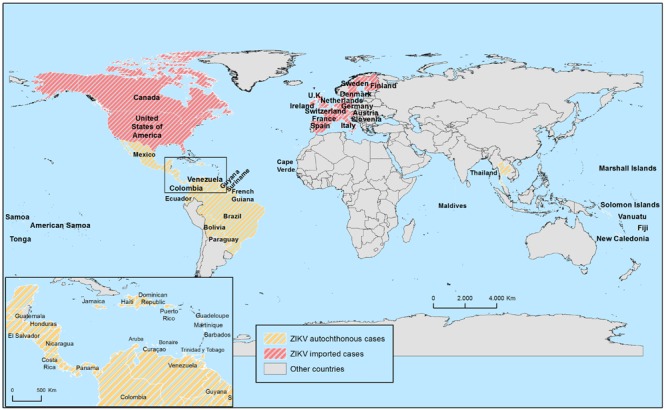
**Map showing worldwide autochthonous and imported ZIKV human cases since the last 9 months.** See text for details.

Since the first isolation of ZIKV in 1954 from an inhabitant of Nigeria (Macnamara, 1954), many serological and entomological studies have reported the circulation of the virus across a widespread area of Africa, including Kenya (Geser et al., 1970), Nigeria (Lee and Moore, 1972; Monath et al., 1973; Fagbami, 1979), Sierra Leone (Robin and Mouchet, 1975), Gabon (Jan et al., 1978; Grard et al., 2014), Uganda (McCrae and Kirya, 1982), Central African Republic (Saluzzo et al., 1981), Senegal (Monlun et al., 1993; Diallo et al., 2014; Althouse et al., 2015), and Ivory Coast (Akoua-Koffi et al., 2001), with prevalence ranging 1.3–52%. At present, Cape Verde is the only African country where, since last year, active viral transmission is currently being reported^8^,^9^ (**Figure 5**). From the beginning of the outbreak in October 2015 to 7th February of this year, the Health Authorities have reported 7362 cases without associated neurological disorders^10^. In any case, and although until now human cases of ZIKV related disease have only been sporadically documented in Africa, it should be kept in mind that this might have been partially due to underdiagnoses, mainly in areas where DENV and CHIKV circulate, as infection with all these viruses presents similar clinical signs (Weissenbock et al., 2010; Grard et al., 2014).

### Asia

In Asia, distinguishing ZIKV infection from other arboviral infections (dengue, yellow fever, and other tropical diseases) is also difficult, so that, early epidemiological data should be treated with caution. In the early 50’s, seroprevalence of ZIKV in humans was reported in several countries: India, Malaysia, Philippines, Vietnam, and Thailand with variable rates, 8–75% (Smithburn, 1954; Smithburn et al., 1954; Hammon et al., 1958; Pond, 1963; Marchette et al., 1969). As the time went by, other countries reported seroprevalence in humans: Indonesia (Java island) from 1977 to 1978 (Olson et al., 1981, 1983) and Pakistan in 1980 (Darwish et al., 1983). More recently a few sporadic human cases have been documented: a confirmed case in Cambodia in 2010 (Heang et al., 2012), an infected traveler who returned from Indonesia in 2013 (Kwong et al., 2013), and some several ZIKV positive cases from travelers returning from Thailand during 2012–2014 (Buathong et al., 2015). Very recently, in January 2016, WHO notified a case of ZIKV infection in a traveler coming back to Finland after spending a few months in the Maldives^11^ (**Figure 5**).

### Oceania

In April 2007, a ZIKV outbreak was reported in Yap Island, Micronesia. The way through the virus was introduced is still unknown, but, as commented before, it has been proposed it was due to an infected mosquito or to an asymptomatic person with undetected infection. Serological analysis indicated that 73% of Yap residents had been infected with ZIKV, but not a single hospitalization, hemorrhagic manifestation, or death were reported during the outbreak (Duffy et al., 2009).

After detection of the first case of ZIKV infection in the French Polynesia in October 2013, during the following outbreak, up to an estimated 11% of population was affected (Cao-Lormeau et al., 2014; Musso et al., 2014b). Most of the clinical cases presented low fever, asthenia, wrist and fingers arthralgia, headache, rash, and only one patient presented Guillain–Barre syndrome (GBS) 7 days after laboratory confirmation of ZIKV infection. It is noteworthy to remark that, after this outbreak, the incidence of GBS in French Polynesia increased 20-fold (Oehler et al., 2014). The reasons for this increase are not known yet. Analyses of the circulating virus have shown that it was genetically closely related to the 2007 Yap and the 2010 Camboya strains, which were not associated to a noticeable number of GBS cases.

Following the French Polynesia outbreak in late 2013, subsequent outbreaks occurred in New Caledonia, Eastern Island, and the Cook Islands. In New Caledonia, the first cases of ZIKV infection were imported from French Polynesia in November 2013, and, subsequently, in January 2014, the first autochthonous case was documented, driving the New Caledonia Health Authority to declare an outbreak situation in February 2014. Up to 1385 laboratory confirmed cases were reported (Dupont-Rouzeyrol et al., 2015). The outbreak in Eastern Island also started in January 2014, accounting for 51 confirmed cases (Tognarelli et al., 2016).

In Australia, the first case of ZIKV infection was notified in 2012 in a traveler returning from Indonesia (Kwong et al., 2013). Since then, all cases have been imported from countries affected by the virus. Nonetheless, as *A. aegypti*, the main ZIKV vector, is present mainly in areas of North Queensland, this region is at risk of an eventual local transmission of ZIKV from infected returning travelers^12^. A few imported cases have been confirmed in New Zealand too, but as the mosquito vectors are not commonly found in these territories, the risk of local transmission is so far low^13^.

In the past 9 months, Fiji, New Caledonia, Vanuatu, Solomon Islands, Marshall Islands, American Samoa, Samoa, and Tonga (**Figure 5**) have reported autochthonous cases^8,9^ but only the latter three have ongoing outbreaks^14^.

### America

At the beginning of 2015, first autochthonous transmission of ZIKV in Brazil was reported and, since then, the virus has rapidly spread throughout the Americas (Zanluca et al., 2015). The Brazilian Ministry of Health estimates that between 440 000 to 1 300 000 cases of ZIKV infections may have occurred in 2015 in the country^15^.

Since the initial report in Brazil, during the last 9 months, and as a 18th of February 2016, 29 countries, or territories, of America have also reported autochthonous cases of ZIKV infection: Aruba, Barbados, Bolivia, Bonaire, Brazil, Colombia, Costa Rica, Curaçao, Dominican Republic, Ecuador, El Salvador, French Guyana, Guadeloupe, Guatemala, Guyana, Haiti, Honduras, Jamaica, Martinique, Mexico, Nicaragua, Panama, Paraguay, Puerto Rico, Saint Martin, Suriname, Trinidad y Tobago, Venezuela, and Virgin Islands^8^ (**Figure 5**). Because the epidemic is still spreading in the Americas, it is reasonable to think that more countries will report autochthonous ZIKV infections in the coming months.

Despite outbreaks have occurred mainly in Caribbean countries/territories and central and south America, imported confirmed cases are starting to show in North America: USA and Canada (**Figure 5**). As of today, 82 and 3 imported cases have been reported in these countries, respectively^16^^,^^17^. Although the risk of ZIKV establishment in Canada and northern USA is low because of the absence of the vectors, it cannot be discarded that the virus persists and spreads in these regions causing autochthonous infected human cases, as it has happened with other related flaviviruses like WNV (Martin-Acebes and Saiz, 2012).

### Europe

Nowadays, there is no evidence of autochthonous ZIKV infection in Europe. All reported cases were imported from people returning from affected countries. The first laboratory confirmed case was notified in November 2013 in Germany (Tappe et al., 2014). Since then, from 2015, imported cases have been documented in Norway (Waehre et al., 2014), Italy (Zammarchi et al., 2015), and Germany (Tappe et al., 2015). However, after the recent explosive expansion of the virus in the Americas, many European countries have reported imported cases of ZIKV infection in travelers returning home (**Figure 5**), including Austria, Denmark, Finland, France, Germany, Ireland, Italy, Portugal, the Netherlands, Spain, Slovenia, Sweden, Switzerland, and UK^18^.

As commented above, currently only *A. albopictus* has colonized Europe, mainly the Mediterranean area (Medlock et al., 2012; Kraemer et al., 2015). Since *A. aegypti* is the main responsible of the current transmission of ZIKV in other regions of the world, the actual risk of a ZIKV outbreak in Europe seems to be low. Nonetheless, beside the possible reintroduction of *A. aegypti* in the continent, *A. albopticus* is circulating in the southern regions of the continent and, since this species can also efficiently transmit the virus, surveillance programs should be implemented, mainly during warm seasons. In fact, autochthonous DENV and CHIKV infections in France (La Ruche et al., 2010; Delisle et al., 2015), Croatia (Gjenero-Margan et al., 2011), and Italy (Rezza et al., 2007) have already been documented.

## Clinical Manifestations and Pathogeny

### Humans

Zika virus infection has been reported to be symptomatic only in around 18% of the cases (Duffy et al., 2009), in which it causes a mild, self-limiting disease with an incubation period of up to 10 days, often mistaken with other arboviral infections like dengue or chikungunya. Clinical manifestations in symptomatic cases resemble that of an influenza-like syndrome (Macnamara, 1954), being the most common symptoms fever, rash, arthralgia, and conjunctivitis; with headache, vomiting, edema, and jaundice being reported less frequently (Zammarchi et al., 2015). Digestive complications (abdominal pain, diarrhea, and constipation), mucous membrane ulcerations (aphthae), and pruritus are rarely observed. The symptoms usually resolve spontaneously after 3–7 days, but arthralgia may persist for up to 1 month (Foy et al., 2011). A post-infection asthenia seems to be also frequent. In any case, severe disease requiring hospitalization has been uncommon until now.

First description of clinical manifestations were reported in 1954 by Macnamara (Macnamara, 1954). In 1956, Bearcroft described the symptoms in an experimentally infected human volunteer (Bearcroft, 1956). A slight generalized headache and a rise in temperature started at the 3rd day, increasing during day 5. By day 7 the patient felt well and the temperature had fallen to normal. Jaundice did not develop, and no other evidence of hepatic dysfunction was found. The total white blood cells during the first 12 days after inoculation did not differ greatly from pre-inoculation counts. Urine estimations were consistently negative for bile pigments and albumin during this period. Estimations of total serum bilirubin carried out between the time of inoculation and the 17th day showed normal levels, and the site of inoculation appeared healthy.

In 1964, Simpson described his own acquired ZIKV illness while working with ZIKV strains isolated from *A. africanus* collected during 1962–1963 (Simpson, 1964). The illness began with a slight frontal headache, showing no other symptoms at the time. During the 2nd day he presented a diffuse pink maculopapular rash which covered the face, neck, trunk, and upper arms that spread gradually to involve all four limbs, and felt slight aching sensations in his back and thighs. Oral temperature at this time was normal in the morning, however he was slightly febrile throughout the day. The temperature returned to normal by the end of the day, and he felt much better, apart from a slight headache. The rash persisted to day 5, when it was fading until completely disappear. No other signs or symptoms were noted during the illness.

Information on laboratory tested alterations associated with ZIKV infection are limited, but may include transient leucopenia, with (Kutsuna et al., 2014) or without thrombocytopenia (Kwong et al., 2013), and slight elevation of serum lactate dehydrogenase, gamma-glutamyl transferase, and of inflammatory parameters (C– reactive protein, fibrinogen and ferritin) (Tappe et al., 2014). Serum aspartate aminotransferase (AST) and alanine aminotransferase (ALT) concentrations may or may not be elevated.

#### Guillain–Barre Syndrome (GBS)

An association of ZIKV infection with more severe disease outcomes, such as GBS has been also proposed. GBS is an autoimmune disease causing acute or subacute flaccid paralysis that can even cause death (van den Berg et al., 2013; Dominguez-Moreno et al., 2014), and that it has been previously associated with other flaviviral infections (Puccioni-Sohler et al., 2012). Remarkably, as aforementioned, during ZIKV outbreak reported in French Polynesia the incidence rate of GBS cases was approximately 20-fold higher than expected given the size of the French Polynesia population and its previously established incidence (1–2/100 000 population per year) (Oehler et al., 2014). Likewise, in Colombia, during the ongoing outbreak, 86 cases of GBS have been associated to ZIKV infection^19^. Based on the 600 000 expected Zika infections in the country, up to 1000 cases of GBS could be anticipated. These data point to a worrisome increase in the potential clinical severity of the disease (Roth et al., 2014).

#### Microcephaly

Similarly to GBS, even more disturbing is the astonishing rise in the number of babies born with microcephaly and neurological disorders that have been suggested to be associated with the current ZIKV outbreak in Brazil (Schuler-Faccini et al., 2016). These congenital infections presumably due to ZIKV exposure have been also associated with an increase in vision-threatening findings, which include bilateral macular and perimacular lesions, as well as optic nerve abnormalities in most cases (de Paula Freitas et al., 2016; Ventura et al., 2016a,b). By the end of 2015, Brazil’s Health Ministry reported an unusual spike in reported cases of microcephaly in the northeastern state of Pernambuco, where the affected children’s mothers had been in early pregnancy at around the same time as large ZIKV outbreaks occurred. The Ministry subsequently raised the alarm of a possible link to ZIKV. Although most cases have been described in Pernambuco, they have also been diagnosed in other eight Brazilian states so far (Oliveira Melo et al., 2016). However, it should be noted that neighboring Colombia has reported over 5000 cases of ZIKV in pregnant women and so far, only one documented case of microcephaly in a newborn, and other two with other congenital brain abnormalities have been very recently documented (Butler, 2016).

In any case, this possible association of microcephaly with ZIKV is still a matter of controversy among researchers. The Latin American Collaborative Study of Congenital Malformations (ECLAMC) suggested that the rise in reported cases of microcephaly might largely be attributable to the intense search for cases of the birth defect, and to misdiagnoses, that arose from heightened awareness in the wake of the possible link with ZIKV. However, it should be noted that a successful RT-PCR amplification of the complete ZIKV genome from a fetal brain tissue has been recently described (Mlakar et al., 2016). In this line, an expectant mother presented a febrile illness with rash at the end of the first trimester of pregnancy while she was living in Brazil. Ultrasonography performed at 29 weeks of gestation revealed microcephaly with calcifications in the fetal brain and placenta. After the mother requested termination of the pregnancy, the fetal autopsy revealed microcephaly, with almost complete agyria, hydrocephalus, and multifocal dystrophic calcifications in the cortex and subcortical white matter, with associated cortical displacement and mild focal inflammation. Electron microscopy analysis also revealed spherical virus particles with morphologic characteristics consistent with ZIKV. Likewise, very recently, the ZIKV genome has been detected and sequenced form amniotic fluid samples of two pregnant women whose fetuses where diagnosed with microcephaly, (Calvet et al., 2016).

The apparent risk of microcephaly was enough for the World Health Organization (WHO) to declare a public health emergency of international concern on February 1 (Rubin et al., 2016) that has lately been integrated into risk assessments by the ECDC^4^. Therefore, the most important current milestone in ZIKV investigations is to clearly elucidate the possible relationship of the infection with the development of serious neurological disorders.

### Animal Models

The pathogenicity of ZIKV has been also evaluated in several animal models. Among these, the most widely used has been the mouse model. Moreover, initial isolation of ZIKV was performed by intracerebral inoculation of viral samples into mice (Dick, 1952). On these initial experiments, infectious virus was only recovered from infected brain mice, whereas no infectious virus able to replicate when inoculated in other mice was recovered from non-nervous tissues such as kidney, lung, liver, or spleens, highlighting the marked neurotropism of ZIKV. Infected mice displayed detectable signs of infection about 5–6 days post-infection, time at which virus titers peaked (Dick, 1952). The analysis of the pathological changes observed in ZIKV-infected mice brains revealed various stages of cellular infiltration and degeneration that were also found in the spinal cords. Degeneration of nerve cells, especially in the region of the hippocampus, resulted in an early and marked enlargement of astroglial cells with patchy destruction of the pyriform cells of Ammon’s horn (Weinbren and Williams, 1958; Bell et al., 1971). Microscopy analysis confirmed that the virus replicates in both neurons and astrogial cells (Weinbren and Williams, 1958; Bell et al., 1971). Remarkably, while mice of all ages tested were susceptible to intracerebral inoculations, mice of 2 week of age and over could rarely be infected by the intraperitoneal route. In contrast, mice younger than 2 weeks were highly susceptible to intraperitoneal inoculation of the virus (Dick, 1952). This finding is similar to that reported for other flaviviruses, such as Usutu virus (Blazquez et al., 2015). ZIKV-induced pathological changes are confined to the central nervous system in older animals, but myocarditis (and associated pulmonary oedema) and skeletal myositis can be also found in young (1–5 days old) animals infected with ZIKV (Weinbren and Williams, 1958). However, it has to be considered that only non-mouse-adapted strain of ZIKV seems to induce myocarditis (Weinbren and Williams, 1958). Besides mice, the susceptibility to ZIKV has been also evaluated in other non-primate small animal models, demonstrating that cotton-rats, guinea pigs, and rabbits did not show clinical signs of infection after intracerebral inoculation of a late passage mouse brain virus. Nevertheless, inoculation of low passage ZIKV in guinea pigs resulted in death at 6 days post-infection (Dick, 1952).

The pathogenicity of ZIKV in monkeys seems to be also mild, except from the sentinel Rhesus 766 from which ZIKV was initially isolated that exhibited a slight pyrexia (Dick et al., 1952). Experimentally infected monkeys developed an unapparent infection after subcutaneous inoculation with mouse recovered ZIKV. Only one of five monkeys tested showed a mild pyrexia after intracerebral inoculation, whereas the others showed no signs of infection (Dick, 1952). In any case, it should be noted that all infected monkeys showed viremia during the 1st week after inoculation, as well as induction of specific antibodies about 14 days post-inoculation (Dick, 1952). This induction of viremia in monkeys is supposed to play an essential role for the establishment of the enzootic transmission cycle between non-human primates and mosquitoes.

## Diagnosis

Different arboviral infections can have similar clinical presentations and, thus, their circulation may be underreported if specific diagnostic tools have not been implemented. In the case of ZIKV, diagnosis presents several drawbacks; there is no “gold standard” diagnosis tool, antibodies are frequently cross-reactive between flaviviruses, which limits the use of serology, viral culture is not routinely performed, and, so far, there is no antigenic detection test available (Musso et al., 2015a). At present, the diagnosis of ZIKV infection is mainly made through “in house” molecular (RT-PCR) and serologic [Ig M ELISA and plaque reduction neutralization test (PRNT)] assays, as only very recently molecular commercial tests for ZIKV have been made available^20^,^21^.

### Antibody

An IgM ELISA was developed at the Arboviral Diagnostic and Reference Laboratory of the CDC to detect ZIKV using samples from Yap Island outbreak (Lanciotti et al., 2008). IgM was detectable as early as 3 days after onset of illness, but cross-reaction with other flaviviruses was observed. This cross-reactivity of sera from convalescent-phase patients was more frequent in those from patients with evidence of previous flavivirus infections than among those with apparent primary ZIKV infections, mainly in the case of previous DENV infection (Lanciotti et al., 2008; Duffy et al., 2009). This IgM cross-reactivity has been further corroborated (Zammarchi et al., 2015; Shinohara et al., 2016), so, as in many other flaviviral infections, ELISA positive results should be confirmed, either by testing an acute-phase serum sample collected as early as possible after onset of illness and a second sample collected 2–3 weeks later, or by additional assays, such as PRNT, that have to result in at least a fourfold increase in ZIKV neutralizing antibody titers when compared with that of the other viruses tested^22^.

Recently, a report analyzing the presence of IgG against different flaviviruses among blood donors in French Polynesia, based on the use of recombinant antigens comprising the domain III of the envelope protein of each virus strain, claimed to differentiate between them, as an overall seropositivity rates of 80.3% for at least one DENV serotype, 0.8% for ZIKV, 1.3% for JEV, and 1.5% for WNV were recorded (Aubry et al., 2015). In any case, it should be noticed that ELISA cross-reactivity can also be the result of co-infections with more than one flavivirus that co-circulate in the same regions, as recently reported for two cases (Dupont-Rouzeyrol et al., 2015).

### Nucleic Acid

The diagnosis of ZIKV is at present primarily based on the detection of viral RNA from specimens by means of RT-PCR (Faye et al., 2008; Cao-Lormeau et al., 2014; Grard et al., 2014; Musso et al., 2015a; Zammarchi et al., 2015; Marcondes and Ximenes, 2016; Shinohara et al., 2016). However, as the viremic period is short, direct virus detection should be performed in samples took during the first 3–5 days after the onset of symptoms (Balm et al., 2012).

A specific ZIKV nucleic acid test (NAT) was implemented in routine practice during the French Polynesian outbreak (Musso et al., 2014a) on the basis of protocols implemented to prevent WNV transmission by transfusion in North America. Additional NATs have been developed based on specific Asian and African ZIKV strains targeting either the envelope or the NS5 regions (Duffy et al., 2009; Faye et al., 2013). Likewise, a one-step RT-PCR assay to detect ZIKV in human serum has also been developed (Faye et al., 2008). The assay showed to be rapid, sensitive, and specific to detect ZIKV in cell culture or serum. Nevertheless, since experimentally infected samples were used, it needs to be validated for diagnosis using clinical samples.

All these NATs have several limitations. Because of its nucleotide sequence specificity, NAT cannot be used to screen a wide range of pathogens with one run, being necessary the use of multiple assays if several pathogens are co-circulating in the same area, but multiple testing is expensive and time-consuming (Aubry et al., 2016), and it does not detect all infected blood donations, especially when nucleic acid loads are low and when sera are tested in large pools (Musso et al., 2015a). Therefore, use of alternative source of samples has been proposed. In this sense, the suitability of urine samples for diagnosing ZIKV infection has been recently confirmed, as RNA of the virus is detectable in urine at a higher load and with a longer duration than in serum (10 to >20 days, and >7 days once it become undetectable in serum) (Gourinat et al., 2015; Shinohara et al., 2016). Saliva has also been used as an alternative sample for routine ZIKV RNA detection, showing positivity more frequently than blood samples, but it did not increase the window of detection in contrast to what was reported for urine. Since ZIKV RNA detection was found negative in some saliva samples while positive in blood, saliva cannot replace blood samples, but just help on detection (Musso et al., 2015a). As a result of these data, it has been suggested to collect both blood and saliva samples to increase the sensitivity of molecular detection of viral RNA for Zika fever diagnosis in acute phase. On the other hand, urine sample can be associated at the late stages of the disease. Thus, when detection of ZIKV is of particular importance, using a combination of samples (blood/saliva/urine) is recommended (Musso et al., 2015a).

## Prophylaxis

Currently there are no specific antiviral agents, vaccine, or prophylaxis for ZIKV. Treatment is generally directed to symptom relief with analgesics and anti-pyretics. Therefore, developing an effective, safe, and affordable ZIKV vaccine, and search for antiviral effective compounds for disease treatment is a current challenge for Zika disease.

Vaccines for various flaviviruses have been produced during the past years, some of them being already in the market, such as those for YFV or WNV (Dauphin and Zientara, 2007; Ulbert and Magnusson, 2014; Monath and Vasconcelos, 2015). These vaccines have been produced using different strategies, inactivated or live-attenuated viruses, recombinant proteins or peptides expressed in different heterologous systems, recombinant subviral particles, chimeric backbone viruses, or naked cDNA (Ishikawa et al., 2014). Thus, it seems reasonable to think that similar strategies can be applied to ZIKV. For instances, a DNA based vaccine (SynCon, Phamaceutical, U.S.) has been recently produced, which it is expected to go into Phase I trials before the end of 2016. In addition, a global patent of two vaccine candidates (a recombinant vaccine and an inactivated vaccine) for ZIKV has been just filled (Bharat Biotech, India).

Developing effective specific therapies for ZIKV seems much more difficult, as so far, and despite several attempts have been made in the past years, none of such compounds are available against any flaviviral infection (Apte-Sengupta et al., 2014). Along with drugs, antibody mediated protection against ZIKV should also be addressed, as in other flaviviral infections (YF, WN, or dengue) experimental specific antibodies treatment has been some time successful (Roehrig et al., 2001; Ben-Nathan et al., 2003; Engle and Diamond, 2003).

Nevertheless, and despite the great effort that probably will be made within the scientific community in the coming years, it will take time until any drugs or vaccines against ZIKV will be commercially available.

### Public Health and Preventive Measures

As previously detailed, ZIKV infection generally causes a non-severe disease (Zammarchi et al., 2015), but some areas newly affected by the virus are providing worrisome information on the already mentioned potential association of neonatal malformations (microcephaly) and GBS with ZIKV (Oehler et al., 2014; Mlakar et al., 2016; Tetro, 2016). As a consequence, and in the absence of another explanation for these connections, the WHO declared a Public Health Emergency of International Concern on the 1st of February of 2016^23^, highlighting the importance of enhance the measures to reduce the ZIKV infection, particularly among pregnant women and women of childbearing age. In this way, as previously remarked, additional investigations are absolutely necessary to determine whether there is a casual link between ZIKV and microcephaly and GBS.

Currently, no vaccine or treatment exists to prevent ZIKV disease, although more probably several vaccine prototypes will be obtained soon. Thus, to facilitate the surveillance and control measures, research and development efforts should be intensified for ZIKV vaccines, therapeutics, and improved new diagnostics^23^, especially if the connection between ZIKV and microcephaly is confirmed.

In the meantime, prevention, and control of ZIKV infection are mainly focused on avoiding the bites of carrying mosquitoes responsible for disease transmission. These measures are the same as those recommended to prevent other diseases transmitted by mosquitoes bite (Martin-Acebes and Saiz, 2012), and include the use of insect repellent, wearing long-sleeved shirts and long pants, the elimination of standing water where mosquitoes can lay eggs, the minimization of outdoor activities coincident with the maximum activity of mosquitoes, the installation of window and door screens, and the implementation of accurate mosquito control programs^24^. ECDC and CDC recommend those pregnant women and those who are trying to get pregnant consider not travel to areas affected by the virus24,^25^. Furthermore, some American countries, including Colombia^26^, Honduras^27^, Ecuador^28^, El Salvador^29^, and Jamaica^30^ have recommended delay pregnancy during the ZIKV outbreak.

On the other hand, in territories where autochthonous ZIKV infection has not yet been detected, but potential vectors are present, the public health institutions should have a quick response to prevent local transmission when an imported case is confirmed. In this context, WHO called for health authorities to collaborate with the transport sector to ensure disinfection of aircraft from affected areas^23^.

As described before, a few cases of sexual transmission of ZIKV has been reported (Musso et al., 2015b; McCarthy, 2016). Even though further studies are needed to confirm the actual sexual risk of ZIKV transmission, the CDC recommend the proper use of preventive measures, such as condoms, if having sex (vaginal, anal, or oral) with a male partner while traveling, or if he has just comeback from an area where the virus is actively circulating; even more, in this latter case, sexual abstinence is recommended if the partner is pregnant^31^.

Likewise, and since spread of the virus through blood transfusion has been reported (Musso et al., 2014a), the countries affected by the virus should take measures to prevent this way of infection (Marano et al., 2016). Moreover, in territories free of ZIKV, people returning from affected areas should delay blood donations^32^.

The rapid spread of ZIKV in Brazil and the Americas, with the suspected neonatal malformations associated, is being cause for alarm and the mass media are even questioning if the 2016 Olympic Games in Rio should be delayed or even canceled. In fact, the Brazilian authorities are performing intense vector control programs to eliminate mosquito vector that transmit ZIKV and other arboviruses (Petersen et al., 2016).

## Final Remarks

After the discovery of ZIKV in 1947, several investigations were performed during the 1950s and 1960s to characterize the new pathogen but, since then, little more was done until the spread of the virus to the Micronesia in 2007. After that, with the explosive invasion of the Americas in 2015, and the possible association of ZIKV with severe neurological diseases, these investigations have significantly increased, and it should be expected that much more information regarding the biology and the clinical consequences of ZIKV infection will be available soon. Several issues have to be resolved. For instance, most of the virus molecular biology and the virus-host cell interactions have been inferred from other related flaviviruses, and, thus, they should be specifically analyzed. Likewise, the elucidation of the presence of determinants of virulence/pathogenicity, and the role that protein glycosylations can play on it will be of much interest. On the other hand, the role of humans in the spread of ZIKV, and whether vertical and sexual transmission routes are important factors for human-to-human spread also need to be clearly determined. No least is the improvement of more accurate diagnostic tools, the development of vaccines, and the design of antiviral therapies. Finally, without any doubt, currently the most important and pressing issue is to reveal whether ZIKV infection is the cause of the increase in the number of GBS and microcephaly cases lately reported.

## Author Contributions

All authors listed, have made substantial, direct and intellectual contribution to the work, and approved it for publication.

## Conflict of Interest Statement

The authors declare that the research was conducted in the absence of any commercial or financial relationships that could be construed as a potential conflict of interest.

## Footnotes

1 http://www.ictvonline.org/virustaxonomy.asp

2 http://ecdc.europa.eu/en/healthtopics/vectors/vector-maps/Pages/VBORNET_maps.aspx

3 http://ecdc.europa.eu/en/healthtopics/zika_virus_infection/factsheet-health-professionals/Pages/factsheet_health_professionals.aspx

4 http://ecdc.europa.eu/en/publications/Publications/rapid-risk-assessment-zika-virus-first-update-jan-2016.pdf

5 http://www.dallascounty.org/department/hhs/press/documents/PR2-2-16DCHHSReportsFirstCaseofZikaVirusThroughSexualTransmission.pdf

6 http://www.paho.org/hq/index.php?option=com_content&view=article&id=11605:2016-paho-statement-on-zika-transmission-prevention-&Itemid=41716&lang=en

7 http://ecdc.europa.eu/en/press/news/_layouts/forms/News_DispForm.aspx?List=8db7286c-fe2d-476c-9133-18ff4cb1b568&ID=1348

8 http://ecdc.europa.eu/en/healthtopics/zika_virus_infection/zika-outbreak/Pages/Zika-countries-with-transmission.aspx

9 http://www.cdc.gov/zika/geo/active-countries.html

10 http://www.minsaude.gov.cv/index.php/documentos/cat_view/34-documentacao/72-zika-virus

11 http://www.who.int/csr/don/8-february-2016-zika-maldives/en/

12 http://www.health.gov.au/internet/main/publishing.nsf/Content/ohp-zika-health-practitioners.htm

13 http://www.arphs.govt.nz/health-information/communicable-disease/dengue-fever-zika-chikungunya#.VsLrOyuG-WM

14 http://smartraveller.gov.au/bulletins/zika_virus

15 http://portalsaude.saude.gov.br/images/pdf/2015/dezembro/09/Microcefalia---Protocolo-de-vigil--ncia-eresposta---vers--o-1----09dez2015-8h.pdf

16 http://www.cdc.gov/zika/geo/united-states.html

17 http://www.healthycanadians.gc.ca/publications/diseases-conditions-maladies-affections/risks-zika-virus-risques/index-eng.php

18 http://ecdc.europa.eu/en/healthtopics/zika_virus_infection/zika-outbreak/Pages/epidemiological-situation.aspx

19 http://www.who.int/csr/don/12-february-2016-gbs-colombia-venezuela/en/

20 http://www.euroimmun.co.uk/recent-news/first-commercial-antibody-tests-for-zika-virus-diagnostics

21 www.genesig.com/assets/files/zikv.pdf

22 http://www.cdc.gov/mmwr/volumes/65/wr/mm6503e3.htm

23 http://www.who.int/mediacentre/news/statements/2016/1st-emergency-committee-zika/en/

24 http://www.cdc.gov/zika/prevention/index.html

25 http://ecdc.europa.eu/en/healthtopics/zika_virus_infection/factsheet-health-professionals/Pages/factsheet_health_professionals.aspx

26 https://www.minsalud.gov.co/sites/rid/Lists/BibliotecaDigital/RIDE/DE/DIJ/Circular-02-de-2016.pdf

27 http://www.salud.gob.hn/

28 http://www.salud.gob.ec/ministerio-de-salud-refuerza-recomendaciones-a-mujeres-embarazadas-por-virus-zika/

29 http://www.salud.gob.sv/novedades/noticias/noticias-ciudadanosas/348-enero-2016/3275--25-01-2016-sistema-nacional-de-proteccion-civil-realiza-jornada-municipal-contra-zancudo-transmisor-del-dengue-chikv-y-zika.html

30 http://moh.gov.jm/presentation/notes-for-minister-of-health-hon-horace-dalley-post-cabinet-press-briefing-january-20-2016-at-11a-m-office-of-the-prime-minister/

31 http://wwwnc.cdc.gov/travel/notices/alert/zika-virus-central-america

32 http://www.msssi.gob.es/gabinete/notasPrensa.do?id=3900
